# Effect of maternal depression on infant-directed speech to prelinguistic infants: Implications for language development

**DOI:** 10.1371/journal.pone.0236787

**Published:** 2020-07-30

**Authors:** Christa Lam-Cassettari, Jane Kohlhoff

**Affiliations:** 1 Western Sydney University, Sydney, Australia; 2 MARCS Institute for Brain, Behaviour & Development, Sydney, Australia; 3 Karitane, Carramar, NSW, Australia; 4 University of New South Wales, Kensington, NSW, Australia; Max-Planck-Institut fur Kognitions- und Neurowissenschaften, GERMANY

## Abstract

The nature and timing of caregivers’ speech provides an important foundation for infant attention and language development in the first year of life. Infant-directed speech is a key component of responsive parent-infant communication that is typically characterised by exaggerated intonation and positive affect. This study examines the effect of postnatal depression on the expression of positive vocal affect and pitch, the quantity of mothers’ infant-directed speech input and the timing of vocal responses between mother and infant. Postnatal mothers currently experiencing symptoms of depression (*n* = 13) were matched to postnatal mothers who were not experiencing symptoms of depression (*n* = 13), and audio-recorded while playing with their 6-month-old infants. Compared with depressed mothers, non-depressed mothers used a higher mean pitch and pitch range, spoke more, gave faster verbal responses and were rated as expressing more positive valence in their voice. These preliminary findings indicate that mothers experiencing low mood use less infant-directed speech and less exaggerated pitch with prelinguistic infants. Postnatal depression is a major health issue that adversely impacts the parent and child. Early interventions for PND may benefit from identifying ways to support the timing of conversations and mothers’ use of appropriate vocal pitch and infant-directed speech modifications. Further research is needed to confirm whether these strategies support early conversations.

## Introduction

Before infants utter their first words, they are learning the protocols of social interaction through the daily experiences they have with their caregivers [1]. Spontaneous social exchanges play an important role in scaffolding early speech. Infant-directed speech (IDS) is a key component of face-to-face interactions in early infancy and is produced (mostly unconsciously) by caregivers in the presence of an infant [see 2,3]. IDS is characterised by a sing-song pitch, generally carried by an exaggerated prosody (fundamental frequency) in IDS compared to the more monotone style used to talk to other adults [3,4]. The exaggerated pitch in IDS functions to communicate affect, promote social interaction [3,5,6], and engage and sustain infant attention [4,7]. The importance of IDS has been demonstrated empirically in studies showing that infants who are not exposed to highly intonated IDS engage less in sustained social interactions; and that infants who experience less IDS input show poorer language development by the second year of life [8–10]. Neurophysiological studies provide further evidence that exposure to prosodic exaggerations in IDS is positively associated with early communicative development. For example, one study showed that experience with responsive maternal interactions including IDS with heightened vocal emotion scaffolds general learning mechanisms in young infants [11]. Another showed that by 7-months of age, infants that are exposed to happy emotional prosody show increased neural responses in a voice-sensitive region in the right hemisphere of the brain [12]. Thus, experience with prototypical IDS prosody and vocal affect not only elicits greater responsiveness from the infant [13], but also fosters a positive interactive loop of social interaction between mother and child [2,6,14] which in turn scaffolds infant vocal communication [15–18], and supports healthy brain development.

Postnatal depression (PND) is a mild to moderate non-psychotic depressive episode, with an estimated prevalence of 10–13% in high-income countries [19,20], and 20% in low- to middle-income countries [21,22]. PND is a global public health issue [21] and leading source of poor health in Australian women [23]. It is well known that PND places significant burden on sufferers, in addition to negatively impacting on parenting capacity [24] and on infants in physical, emotional and cognitive domains [25]. While genetic and epigenetic factors have been implicated as mechanisms linking PND with poor child outcomes, lowered maternal sensitivity due to maternal depression symptoms are thought to be key [25].

The quality of maternal vocalisations are known to be a key element of maternal sensitivity [26–28], however, only a few studies have specifically examined links between IDS and PND. To understand the extent to which acoustic characteristics of IDS are affected by the emotional state of the mother, Bettes and colleagues studied a sample of 36 postnatal mothers with self-reported depression symptoms and showed them to have a flatter pitch when speaking to their infants at 3–4 months postpartum [29]. Mothers with elevated depression symptoms also displayed slower responses to infant vocalisations [29]. In 2001 Kaplan and colleagues similarly observed a flattened vocal pitch among a sample of 50 mothers at 4–12 months postpartum when mothers were asked to encourage the infant to play with a soft toy using the phrase “pet the gorilla” [30]. Notably, mothers in remission from PND expressed a similar fundamental frequency (pitch) to non-depressed mothers [30], which indicates that the effects of PND on IDS can be reduced when depressive symptoms are reduced. Most recently, in a sample of 281 families with infants aged 3–14 months, Porritt and colleagues [31] showed that mothers with clinically diagnosed PND produced a smaller pitch range than mothers diagnosed with PND in partial remission, and that deficits in pitch range were not well predicted by elevated self-report scores alone, or by diagnosed anxiety disorders. Together, the studies by Bettes et al. [29], Kaplan et al. [30] and Porritt et al. [31] are important because they demonstrate links between PND and diminished pitch variation, particularly in IDS within structured play contexts when lexical content and the type of interaction are afforded greater control.

There is also evidence of differences in the content of the IDS words spoken between mothers with, and mothers without, PND. Herrera and colleagues recorded maternal speech to 6- and 10-month-old infants and classified it into two categories: affect-salient (content words were expressive of feelings) or information-salient (content was object-oriented) [32]. In this study, mothers experiencing depression produced less affective and informative lexical content while speaking to their 6-month old infants, compared to control mothers. Furthermore, PND mothers’ affective speech content showed little change from 6 to 10 months [32], indicating that mothers were less likely to talk about feelings and adapt their speech to infant developmental needs compared to their non-depressed counterparts.

Taken together, the evidence suggests that American and British mothers experiencing PND speak to their infants with less vocal pitch variation [29–31], poorer temporal responses to infant cues [29] and fewer affective or informative words in IDS [32]. There have been no studies, however, that have compared mothers with, and mothers without, PND in terms of the quality of *affective intent* of IDS, a distinguishing IDS feature known to support mother-infant social interaction and express communicative intent [33]. In normative samples, the affective quality of IDS has been studied. Mothers have been shown to adapt the expression of affect in IDS, with their speech judged by naïve raters as sounding predominately “comforting or soothing” at birth, “approving” at 6 months and “directive” around 9 months [5]. Notably, infant preferences for affective intent adapt across the first year of life, and infant responses have been shown to match the affective intent types that dominate mothers’ speech at 3, 6 and 9 months [6]. Given that mothers’ adaptive use of IDS prosody and affective intent appear to support infants’ predisposition to attend to IDS, it is vital that IDS of mothers with PND is empirically examined not only in terms of pitch modifications and affective content, but also with respect to the expression (and perception) of vocal affect.

A final aspect of IDS that has not been well investigated in PND samples is that of vocal turn taking. ‘Synchrony’, a term often used to describe the interactive and attuned ‘dance’ between a mother and infant, expressed in coordinated exchanges of emotion, facial expression, bodily movements and vocal turn taking, is known to play a vital role in healthy infant development [34,35]. While there have been many studies examining vocal turn taking in mothers and young infants [36,37], little is known about vocal turn taking in the IDS of mothers with PND. In one study, mothers with elevated PND symptoms were shown to display less affective and behavioural synchrony with their infants than non-symptomatic mothers [38] but more work is required to better understand how the experience of PND influences the quality and timing of mother-infant vocalisations.

Gaining better understanding the effects of PND on IDS pitch, vocal affect and mother-infant vocal turn-taking behaviours will provide new insight into components of the early social environment that would benefit from increased support to mitigate the effects of PND before infants develop poor outcomes. To this end, the current study compared acoustic, perceptual, temporal and quantitative measures of IDS (pitch, ratings of vocal affect, number of words, and vocal response timing), and the quantity of infant vocalisations in infants aged around 6-months. Infants aged 6 months were studied because the first 6 months is when infants typically spend most of their waking time with maternal caregivers who may be experiencing the negative symptoms associated with PND. The first 6 months are also a period when IDS pitch exaggerations are most pronounced [5], and the pattern of turn-taking cues that mothers provide shape the emergence of babble [39]. Critically, there is evidence that the timing of mothers vocal responses influences the complexity of infant babble before first words emerge [15,16] thus providing a foundation for later language development [40]. Given previous evidence of flatter pitch [29,31], and less affective content words [32] among mothers with PND, we hypothesised that compared to mothers without PND, mothers with PND would show reduced acoustic quality (pitch exaggeration), a reduced number of vocalisations and less positive vocal affect, and a difference in the timing of vocal responses following infant vocalisations. Since maternal speech input has been shown to influence infant babble [15,16,39], we hypothesised that the frequency of infant vocalisations would emerge later due to less exposure to IDS.

## Materials and methods

All procedures were approved by the Human Research Ethics Committee at Western Sydney University and South West Sydney Local Health District. In accordance with the ethics approval, all mother’s provided written and oral consent prior to participating in the study with their infant.

### Participants

Participants were 26 mothers and their infants (*n* = 13 PND group, *n* = 13 non-PND group), recruited from two sites) the Karitane residential parenting centre, a 4 to 5 day residential parent-infant program for unsettled infant behaviour in Sydney Australia, and 2) MARCS Institute BabyLab at Western Sydney University. Four infants in the PND group were recruited through the MARCS Institute BabyLab site after scoring >12 on the Edinburgh Postnatal Depression Scale and having an infant aged 4–7 months. Participants from the Karitane residential parenting program sample were selected for the current study if the mother scored >12 on the Edinburgh Postnatal Depression Scale (EPDS) [41] and the infant was aged 4–7 months. The EPDS is a 10-item questionnaire originally developed to assist in identifying possible symptoms of depression in the postnatal period. It is routinely used in antenatal and perinatal care in Australia and has adequate sensitivity and specificity to identify depressive symptoms using a research cut-off > 12 [42]. Mothers rated how they felt in the previous 7 days from “as much as I always could” to “not at all”. Participants from the MARCS Institute BabyLab sample were selected for the current analysis as the non-PND control group (matched for infant age and sex) if the mother scored < 8 on the EPDS. All mothers were primary caregivers, spoke English, were primarily Caucasian in appearance and resided in the Sydney metropolitan area; infants were primarily first born and reported to be healthy with no remarkable medical conditions. Additional characteristics of the sample are shown in Table 1. It should be noted that two additional mother-infant pairs were tested in the PND group, but one mother intermittently hummed and did not produce spontaneous speech, and another did not produce any speech during the play session, thus were excluded from the PND sample and were not matched to a non-PND dyad.

**Table 1:** Demographic data for mother-infant participants for the PND and non-PND group.

	PND (n = 13)	Non-PND (n = 13)
	*Mean* (SD)	*Mean* (SD)
**Maternal Age (years)**	32 (5.7)	32 (4.7)
**18–24**	2	0
**25–31**	4	7
**32–38**	5	4
**39–44**	2	2
**EPDS Total Score**	14.8 (1.8)	2.8 (2.8)
**0–4**	0	10
**5–8**	0	3
**9–12**	0	0
**13–16**	8	0
**16–20**	5	0
**Infant Age (weeks)**	23 (5.2)	25 (1.89)
**Infant Age Range (weeks)**	15–30	22–29
	*n*	*n*
**Mothers’ living situation**		
**Married/Defacto**	10	13
**Single parent**	3	0
**Caucasian**	10	11
**Other**	3	2
**SRI medication**	2 (Citalopram)	0
**Infant Sex**		
**Female**	6	6
**Male**	7	7
**Infant birth Order**		
**1**	9	9
**2**	4	3
**3**	0	0
**4**	0	1

### Procedure

Mother-infant dyads were audio-recorded during an unstructured free play session in a quiet room at MARCS Institute BabyLab or the Karitane residential parenting centre. Unstructured free play was used to provide caregivers with ample opportunity to spontaneously interact with their child and choose how they play with the available toys. For the free play session, a small selection of age appropriate toys and books were made available for parents and the mothers were asked to “play and spend time with (baby’s name) as you normally would at home” for approximately 5 minutes.

### Data extraction for acoustic analyses

To ensure acoustic analyses were performed on IDS containing active vocal interaction from across the play session [29], a series of audible IDS utterances were concatenated into a 30 second sample for each mother. Speech was segmented from the 2nd, 3rd and 4th minute of the interaction (after reducing silences >500ms, segments that were sung, whispered, interrupted or overlapped with non-speech sounds e.g., clapping, or patting toys) until 10-seconds of vocalisations was extracted for each mother using Praat Acoustical Analysis computer software [43]. Thus a global measure of fundamental frequency (F0) was calculated from clear and audible utterances extracted from the beginning of the first utterance, to the offset of the utterance in each 10 second segment. Mean F0 was averaged over the pitch cycles between onset and offset markers, and range F0 was defined as the difference between the maximum and minimum F0 points between the onset and offset markers for each segment.

### Frequency of vocalisations and maternal temporal responses

The frequency of vocalisations for mother and infant were annotated manually in Praat software to extract the number of words spoken by the mother and the infant across the unedited mid 3-minutes of the 5-minute free play session. The first minute of the play session was excluded because many of the mothers reiterated variations of the instruction that they were given “to play” with their baby to avoid inflating the vocalisation count for both groups. The last minute was excluded from the vocalisation counts because some infants became increasingly fussy (3 from the PND group, 4 from the non-PND group), or their mothers stopped talking during the 4^th^ minute of interaction (3 mothers in the PND sample). The frequency of infant vocalisations included all vocal attempts (cries, vowel sounds, or babble), but excluded breathing sounds [29], because early speech like vocalisations provide the foundation for later speech production [44].

Maternal speech response was defined as the time it took a mother to respond to each infant vocal attempt (in milliseconds) in the 3-minute audio recording used to extract vocalisation counts. Maternal response time was annotated as a “response” if it occurred within 2 seconds of the offset of the infant’s last vocalisation, following evidence that responses that occur within 2-seconds provide optimal support to social interactions [45]. Occasions in which mothers’ vocalisations began prior to the offset of the infant vocalisation were classified as overlap, and an overlap count was recorded.

Interrater reliability checks were performed on 50% of randomly selected cases by a rater masked to participant group and specific hypotheses of the study. Reliability estimates (kappa) were .96, .84, .84 and .91 for maternal word count, infant word count, maternal response time and overlapping speech count, respectively.

### Affective intent

**Fig 1:**
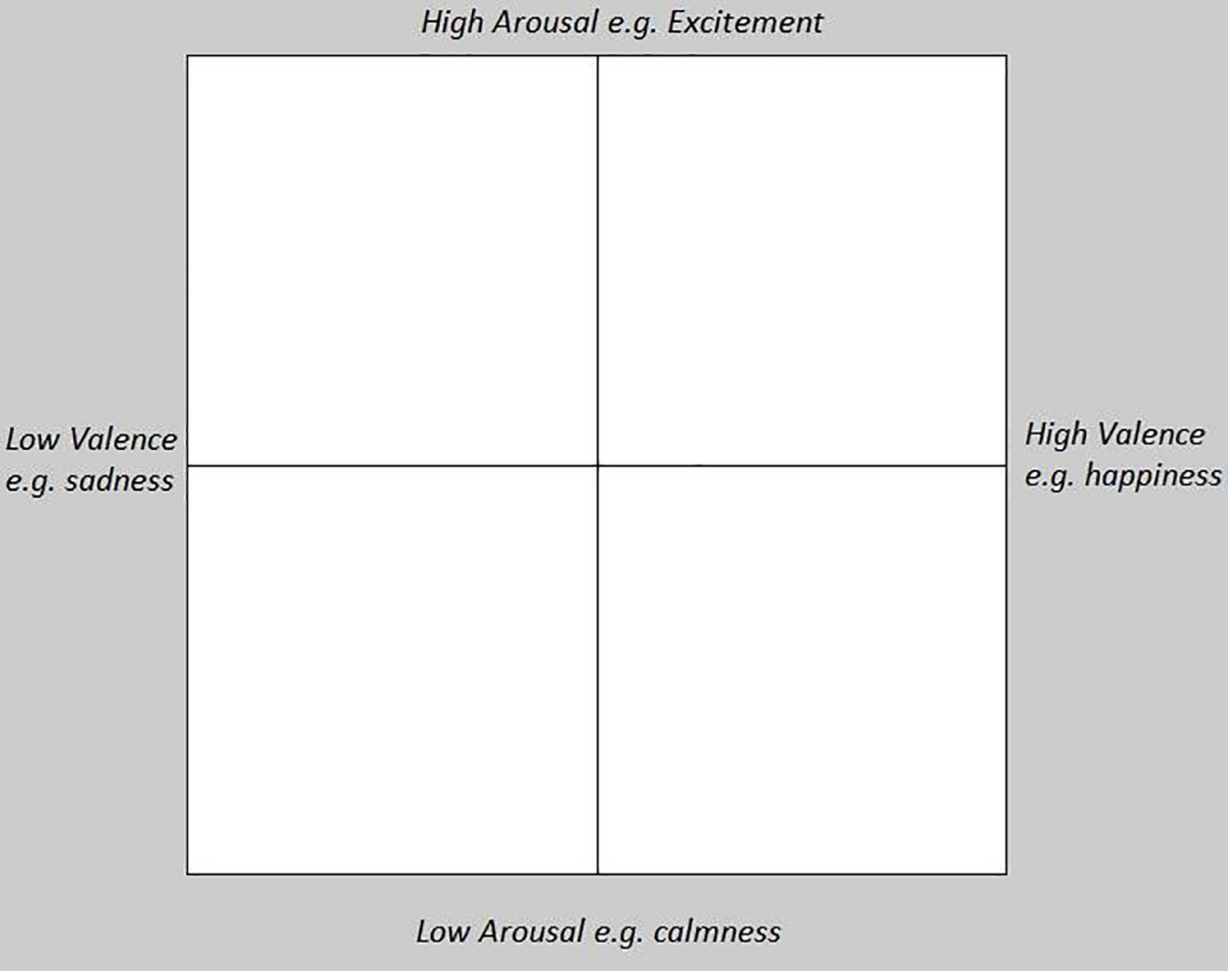
The 2DES software in use, participants move the mouse cursor across the x-y axis to rate the level of arousal and valence.

In line with previous IDS affect ratings studies [5,46], following the initial 60-second warm-up period 25 seconds of speech was sampled from each recording by extracting and concatenating each successive utterance that did not contain background noise or non-speech sounds (e.g., microphone bumped, clapping etc.); and reducing long pauses to 1.5 seconds to remove any prolonged silences from the speech sample. To ensure the segmental content of the speech samples would not influence participant ratings, speech recordings were low-pass filtered at 450 Hertz in Praat [43] deeming the content unintelligible while leaving the prosody, perceived as pitch, intact. Twenty-three undergraduate Psychology students at Western Sydney University rated the low-pass filtered speech samples for affective intent. The students received course credit toward their 1st year Psychology subject in return for completing the ratings task. The majority of raters were female (83%) and born in Australia (70%; n = 4 born in south east Asia, n = 3 born in the Middle East). They had a mean age of 21 years (range 18–33 years) with an average of 4 years experience caring for children <5 years of age at study participation (range 0–14 years experience of caregiving). All raters had normal or corrected-to-normal visual acuity, and did not report any hearing loss. Ratings were completed using a laptop computer installed with MARCS Institute BabyLab’s 2 dimensional emotional space (2DES) software, that indicates the level of valence (affect) and arousal in the IDS recordings (see Fig 1). Ratings were made after listening to low-pass filtered IDS examples exhibiting different levels of affective intent [6] through headphones (so ratings were based on intonation and not semantic content). All raters completed four practice trials before the 26 test trials (one from each mother). The 2DES rating software automatically randomised stimulus presentation for each participant. Ratings were made by moving the mouse cursor around an x-y axes for the duration of the trial (~25 seconds) to indicate the level of i) *Valence* on the x-axis (from low negative = *sadness* to high positive = *happiness*), and ii) *Arousal* on the y-axis (from low = *calmness* to high = *excitement*). Ratings took approximately 20 minutes. Ratings of affective intent for each mother were averaged across raters for the emotional valence and emotional arousal scale.

### Data analysis plan

Inspection of the demographic characteristics detailed in Table 1 indicated that PND versus non-PND groups were comparable in terms of infant age, sex or parity, maternal age, cultural appearance, and relationship status. Planned comparisons [47], defined a priori and independently of the data, were performed using two tailed independent samples t-tests not corrected for multiple comparisons to test our hypotheses that: mothers with PND would produce constraints in measures of vocal F0, the quantity of IDS, quantity of infant vocalisations and mother’s vocal response time to infant vocalisations compared to mothers without PND. An independent samples t-test was used to test whether ratings of positive vocal affect were greater for mothers in the non-PND compared to PND group. Summary data underlying the acoustic measures, vocal counts, response times and ratings of vocal affect are available from https://doi.org/10.26183/5e267dafc9cad.

## Results

### F0 acoustic analysis

Compared to mothers in the non-PND group, mothers in the PND group were shown to express a statistically significant difference in mean F0 (*t* = 2.22, *df* = 24, *p* = 0.036, Cohen’s *d* .*86*), maximum F0 (*t* = 4.40, *df* = 24, *p* = 0.01, Cohen’s *d 1*.*73*) and F0 range (*t* = 4.40, *df* = 24, *p* = 0.01, Cohen’s *d 1*.*73*). Mean F0, maximum F0 and F0 range were lower in mothers experiencing PND symptoms than non-PND mothers (as shown in Table 2). There was no difference between groups in terms of minimum pitch (*p* = .09, Cohen’s *d 0*.*03*).

**Table 2:** Descriptive statistics, statistical results, significance levels and 95% confidence intervals are shown for measures of fundamental frequency (F0), word counts, and vocal response latency in the PND-group and non-PND group with n = 13 in each group.

	NON-PND Group Mean (SD)	PND Group Mean (SD)	t	p	95% CI
**Mean F0 IDS (hertz)**	289 (28)	251 (56)	2.22	.036*	2.73, 73.95
**Minimum F0 IDS (hertz)**	113 (37)	112 (31)	.088	.09	-26.63–28.63
**Maximum F0 IDS (hertz)**	571 (46)	444 (93)	4.40	.01**	67.15, 185.79
**F0 Range IDS (hertz)**	458 (65)	332 (80)	4.40	.01**	66.5, 184.10
**Maternal Word Count**	316 (56)	160 (57)	7.04	001**	110.25, 201.75
**Child Vocal Count**	17 (10)	8 (9)	2.46	.001**	1.45, 16.71
**Maternal Vocal Response Time (ms)**	261 (203)	617 (371)	-3.03	.022*	-597.22, -113.61
**Mother-infant Overlap Count**	9 (4)	4 (6)	2.02	.049*	.017, 10.60

*Indicates statistical significance at *p < 0*.*05*

**Indicates statistical significance at *p ≤ 0*.*01*.

### Frequency of vocalisations and maternal response latency

Compared to mothers in the non-PND group, mothers in the PND group spoke significantly fewer words (*t* = 7.04, *df* = 24, *p* = <0.001, Cohen’s *d 2*.*76*) and their infants made fewer vocalisations (*t* = 2.45, *df* = 24, *p* = .022, Cohen’s *d 0*.*95*). With respect to maternal vocal response timing, mothers in the non-PND group vocalised faster than those in the PND-group following on from infant vocalisations (*t* = -3.03, *df* = 24, *p* = .006, Cohen’s *d -1*.*19*); and non-PND mothers produced more speech overlaps with their infants (*t* = 2.07, *df* = 24, *p* = .049, Cohen’s *d 0*.*98*). Cohen’s *d* calculations indicated large effects for all measures accept minimum F0, which did not differ statistically between groups, as shown in Table 2.

### Affective intent

**Fig 2:**
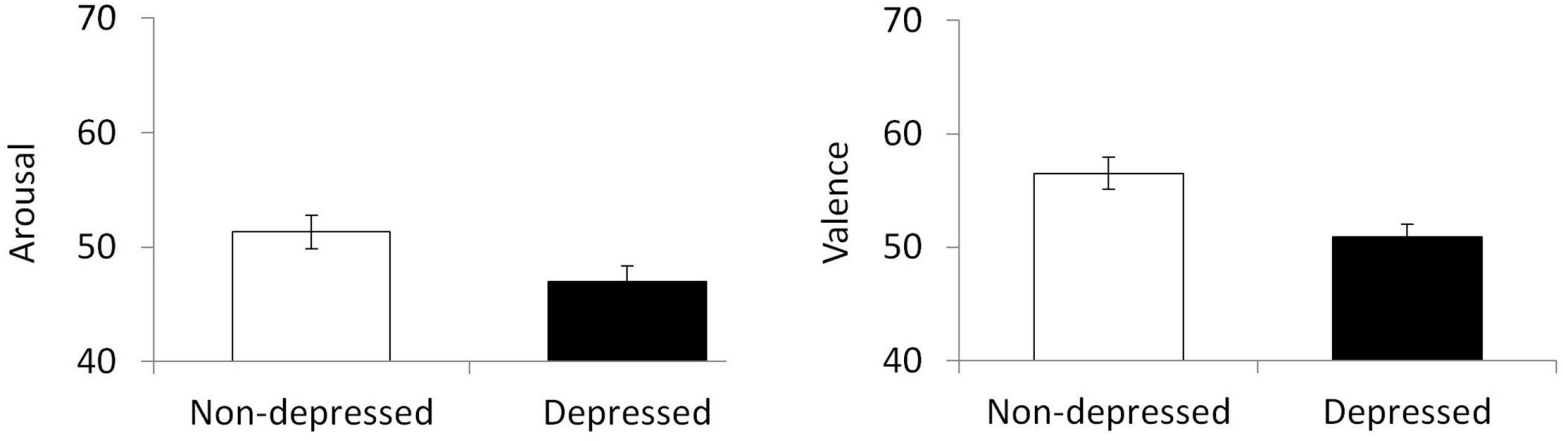
Mean ratings of arousal and valence in mother’s low pass filtered IDS using the 2DES model. Error bars indicate standard error of the mean.

As shown in Fig 2, naïve adults rated IDS affective quality differently for the PND and non-PND groups. Specifically, the IDS of mothers in the non-PND group was perceived to have a larger degree of positive emotional valence (happiness-sadness) (*t* = 4.067, *df* = 22, *p* = 0.001) 95% CI [5.62, 8.31] and greater level of arousal (excitement-calm) (*t* = 3.785, *df* = 22, *p* = 0.001) 95% CI [5.62, 8.31] (see Fig 2).

## Discussion

This preliminary study tested whether measures of vocal pitch (F0), affective intent, number of maternal vocalisations, number of infant vocalisations, and maternal response latency following infant vocalisations differ in IDS of mothers with elevated symptoms of PND and a matched sample without PND. As hypothesised, infants with mother’s experiencing PND had evidently different social interactions to peers with non-PND mothers. Specifically, the vocal environment of 6-month old infants in the PND group showed less pitch variation, a reduction in the expression of IDS positive affect, fewer vocalisations on the part of both mother and infant, less speech overlap, and slower vocal responses by the mother following an infant vocalisation.

Mothers with elevated PND symptoms spoke with a lower F0 and less F0 variation, two features that are exaggerated in prototypical IDS [3,4], and known to engage and maintain infant attention to speech [4,7]. Results of this study concur with findings that American and British mothers with PND speak with a smaller pitch range than non-PND mothers in brief [29] and structured play sessions with 3–14 month old infants [30,31]. They also support previous findings that Australian mothers exaggerate F0- mean and range during short play sessions with 6-month old infants [5,48]. In this study, mothers were audio recorded in a free play session and did not have to “perform” in a structured manner. Thus, despite showing a reduction in F0 at the group level, the F0 range for both groups in the current study are higher than other studies in which mothers were asked to play with a set of provided toys during a “play session” [31,48,49]. Indeed, prior studies instructing mothers to produce a set of target utterances to initiate play show an increase in pitch when mothers ask their infant to “pet” a toy [50], or read three utterances from a book [51]. Despite group differences in mothers F0, it should be noted that the PND mothers did not produce “flat” speech, rather, they produced less exaggerated F0 compared to the non-PND group. Given this is a relatively low-risk sample, we would expect more profound effects in cases of more severe and chronic depression.

This study provides new evidence that mothers experiencing PND symptoms express less vocal energy while engaging in spontaneous play with prelinguistic infants. The quality of affective intent expressed in low-pass filtered IDS samples from mothers with elevated PND symptoms was rated as less *arousing* and expressing less positive *emotional valence* compared to mothers who were not experiencing symptoms of PND. This shows that untrained adults naïve to the mental health of the speaker can hear noticeable differences in mother’s IDS. These findings extend previous evidence that non-PND mothers adapt positive affective intent when speaking with prelinguistic infants [5] and suggest that the heightened positive affect that is carried in IDS is affected not only by infant responsiveness [6], but also by maternal mood state.

Mothers in the PND group said fewer words during free play with their infant, suggesting that mothers experiencing PND provide less communicative input to their infants. This finding is important because exposure to speech, and the level of involvement that infants have with early conversations is associated with early language development [8,10,15,16,18]. Providing prelinguistic infants with sensitively timed conversational turns offers a source of positive reinforcement for infant vocal expressions and engages infants in social interaction [2,3,27,52,53]. Examination of vocal response time revealed that, similar to Bettes (1988), mothers experiencing PND took longer to speak following infant vocalisations compared to the non-PND group. The results also concur with findings that the timing of maternal vocal responses following the offset of child vocalisations is slower with pre-school aged children when mothers experience depression [54]. Interestingly, mother-infant dyads in the PND group produced less speech overlaps, which implies that divergent vocal patterns are emerging in early infancy in relation to the frequency and timing of maternal speech input. The rate of overlap in the non-PND group was comparable to longitudinal evidence that 40% of infant vocalisations occur in overlap with maternal speech between 3–5 months of age [39]. Thus the delayed vocal responses and reduced speech overlaps appears characteristic of the decreased energy associated with PND.

The finding that the number of vocalisations produced by infants of non-PND mothers almost doubled the vocalisations of infants in the PND group contributes new evidence that PND adversely affects early proto conversations [44]. The present study is cross-sectional and so does not directly test whether IDS of PND mothers predicts poor language outcomes. Findings do, however, highlight the importance of evaluating the prelinguistic environment of infants as part of wider care for mothers’ with PND, and confirm the need for further longitudinal research that tests whether decreased vocal affect and pitch modulation in the prelinguistic period is directly related to early language outcomes. Future work should also collect additional socio-economic status information, to directly test the extent to which socio-economic status predicts language development.

The current study had a number of strengths including use of a PND sample of mothers with elevated PND symptoms currently attending a residential parenting centre compared to non-PND controls residing in the same metropolitan area (matched for infant age and sex, and comparable in maternal age, ethnicity, and maternal relationship status). It also extends evidence on the most commonly reported IDS feature F0; provides new evidence that IDS affective intent is less exaggerated by mothers experiencing PND symptoms; and demonstrates differences in the amount of vocalisations, and speech timing of the mother and infant in the prelinguistic period.

A number of study limitations, however, must be acknowledged. First, the sample size was small and recordings were conducted in a laboratory play room or playroom at a parent service, thus results should be interpreted with caution. Second, the population sampled may not be fully representative of the general category of depressed mothers as the majority of the PND group were attending a service for sleep and settling issues. This is important, because the differences in mothers’ speech input observed in this study might be related to aspects of the behaviour of the infants in the PND group rather than the PND symptoms. Finally, the free play sessions were unstructured and mothers were instructed to interact as they “normally would at home”. While it may be argued that a structured task can increase the comparability of the play sessions between groups [11,30,31], the spontaneous nature of the free play was employed to increase the opportunity for mothers to instinctively modulate their vocal pitch and remove any expectations they may have on how much they should speak to their infants. We believe the nature of the play session, thus, contributed to the differences shown in measures of the acoustic and affective quality of IDS, and the number of vocalisations made by the mother and the infant. Future research should heed this design and increase the ecological settings in which research is conducted.

In conclusion, this preliminary study provides new evidence that mothers with elevated PND symptoms express marked differences in IDS to prelinguistic infants; mothers produced less modulation in vocal pitch, less positive vocal affect, and spoke less to their infants during spontaneous free play. Infants of mothers with elevated PND symptoms made less vocalisations with their mothers, who were slower to respond to their vocalisations than non-PND mothers. Our findings suggest mothers experiencing PND symptoms express less IDS modifications in face-to-face interactions in the first half year of life. Notably, these differences were observed in components that have recently been shown to enhance early language abilities in intervention studies with non-PND mothers [8,10], such as modulated IDS pitch and the timing of vocal responses. Given that PND affects many aspects of a person’s behaviours, strategies to increase mother’s energy levels and encourage short, responsive social interactions to encourage increased vocal participation by infants may help minimise negative outcomes associated with reduced interaction in the first year of life. Further research is essential to confirm whether these differences endure, and identify how to best support such strategies in early interventions.
